# A Scoping Review on the Opportunities for Social Engagement and Cognitive Frailty in Older Adults

**DOI:** 10.3389/phrs.2024.1606494

**Published:** 2024-02-08

**Authors:** Sally Fowler Davis, Charlotte Benkowitz, Carol Holland, Alan Gow, Charlotte Clarke

**Affiliations:** ^1^ Faculty of Allied Health and Social Care, Anglia Ruskin University, Cambridge, United Kingdom; ^2^ Centre for Applied Health and Social Care Research, Sheffield Hallam University, Sheffield, United Kingdom; ^3^ Division of Health Research, Centre for Ageing Research, Lancaster University, Lancaster, United Kingdom; ^4^ Department of Psychology, School of Social Sciences, Heriot-Watt University, Edinburgh, United Kingdom; ^5^ Faculty of Social Science & Health, Durham University, Durham, United Kingdom

**Keywords:** social determinants, older adults, population health, cognitive frailty, social engagement

## Abstract

Cognitive frailty (CF) is defined as the clinical syndrome of the combination of physical frailty and cognitive impairment, without dementia. Numerous risk factors for CF have been previously identified but this scoping review focusses on the critical need for social engagement and the association with cognition. The focus of this scoping review on the opportunity for social engagement rather than on perception or experience of loneliness. Based on the results of 55 studies were synthesised into four social engagement categories, namely participation, household, network, and habitat. Social engagement is associated with maintaining or improving cognition, particularly through active participation in social roles. Habitat (i.e., rural or urban settings) also influences cognition and the challenge is to enable social participation.

## Introduction

CF is defined as the conjunction of physical frailty with cognitive impairment but without dementia [1]. Mild cognitive impairment (MCI) has been widely conceptualized as an intermediate phase between cognitive ageing and overt dementia [2]. CF is a phenomenon of older adult population health and may affect 1.0%–12% community-dwelling older adults [3]. CF is differentiated from MCI because it is not necessarily an intermediate stage between normal ageing and dementia and is a potentially reversible condition [3] but can represent deficits including depression, decreased function, social and physical frailty [4].

Behavioural risk factors are common to MCI and CF, including prolonged sedentary behaviours, inactivity, and manifest alongside diabetes and neurological disorders [5, 6]. The Cochrane review, “Prevention of Dementia and Cognitive Decline” [7] suggests that sustained implementation of multi-modal interventions would have a preventative impact at population level, but considerable research is needed to evidence the best diet [8], exercise [9] and prevention strategies [10]. The association between cognitive and physical decline interact [11], resulting in slower cognitive processing speed along with reduced walking speed and grip strength [12].

The World Health Organisation (WHO) Age friendly environments strategy recognises that the loss of physical and cognitive function may relate to environment and reflect limited opportunities for older adults to participate at a community level, where there are inadequate facilities or municipal services [13]. The UN General Assembly declared 2021–2030 highlighted the importance for policymakers to improve the lives of older people [14]. Global variation in expected community participation is reflected in cultural norms for older lifestyles. Intergenerational households may not mitigate cognitive changes, in spite of assumed benefits to safety and company [15]. A consideration of the environmental factors alongside more recognised behavioural/lifestyle interventions is well justified [16].

Targeting health inequalities in relation to CF and identifying environmental risks is important and necessary to achieve the required public health response at community or societal level. CF varies across demographic, social and economic groups in the older adult population and indeed there is considerable variation in reporting of morbidity [14]. Where social activities are well established in communities, they provide instrumental and/or emotional support [17]. Safe and high-quality amenities; inclusive spaces in the neighbourhood may be a requisite factor in enabling social participation [18]. Social engagement has been defined as “the maintenance of social connections and participation in social activities” [19], but it has also been used as an umbrella concept for the variety of components of an individual’s social behaviour and social structure [20]. Older populations are particularly affected by the availability of social activities and pursuits, with variation in cognitive ageing recognised as an outcome [10]. The decline in cognitive health is associated with lower socio-economic status [21]. Both racial and economic disparities also intensify barriers to engagement for more marginalised older people [22].

Compared to previous generations, older adults in democracies with market-based economies (Organization for Economic Co-operation and Development- OECD countries) are active, healthier, wealthier and educated [23]. Networks of family and friends are a source of support and are generally associated with higher life satisfaction values [24] but older adults are also more often single and childless. Factoring in retirement or age-related losses (e.g., death of a partner or friends), along with declining health and increasing mobility limitations, it is more common for older adults to live in relative isolation [25].

The aim of this study was to scope the literature to identify associations between social engagement and cognitive frailty. The review was selected to clarify concepts and make a preliminary assessment of how different contexts and demographic factors related to cognitive frailty.

## Methods

This scoping review follows the guidelines by Arksey and O’Malley [26] that recommend a broad search of the literature on a specific topic which can then be subject to consultation and become useful to policymakers, practitioners, and service users. The research question was “what impact does social engagement have on older adult cognitive frailty?” and searches were undertaken using a specific limited database search that identified relevant studies.

Scopus was selected as a single database based on pragmatic need to complete the study within a timeframe and evidence an inclusive cross-disciplinary literature [27]. The terms cogniti* AND resilience AND “social engagement” and cogniti* AND ageing AND “social engagement” were searched. Scopus pilot searches resulted in the decision to exclude the term “frailty” as they resulted in many findings on physical frailty only (even when including cogniti*) and often did not touch on cognitive decline or impairment. The focus on cogniti* was most useful and enabled review of the findings for age groups/older adults and cognitive frailty in the title and abstract screenings.

The study selection involved i) removing duplicates, ii) title screenings and iii) abstract screening, and using inclusion and exclusion criteria to identify the final selection of studies. Inclusion: the impact of social engagement on cognition, referenced older adults, or reported on observational data or longitudinal cohort investigations, in English. Exclusion: focus solely on physical frailty or dementia, books, reviews and commentaries and if they were associated with lifestyle or behaviour change interventions or particular therapeutic methods that are used to enhance individual capabilities in either healthy or diseased populations.

As suggested by Arksey and O’Malley [26], coding and charting was used to identify multiple types of social engagement, and these were collated into categories which summarised different social environments. Charting was used to synthesise and interpret the coded data into themes according to Ritchie and Spencer’s method in framework analysis [28]. Charting involved sifting and sorting coded texts according to key issues and themes. This was undertaken using the software package Quirkos, that is commonly used in qualitative inquiry, and which allowed the collation and summarising of results. Data regarding the type of study, country, main findings, and type of social engagement were extracted from the papers. The data categories also related to the demographic features of gender and socio-economic status. The outcome is the categorisation of the literature associated with social environments that enable engagement or inhibit social engagement for older adults.

## Results

**FIGURE 1:**
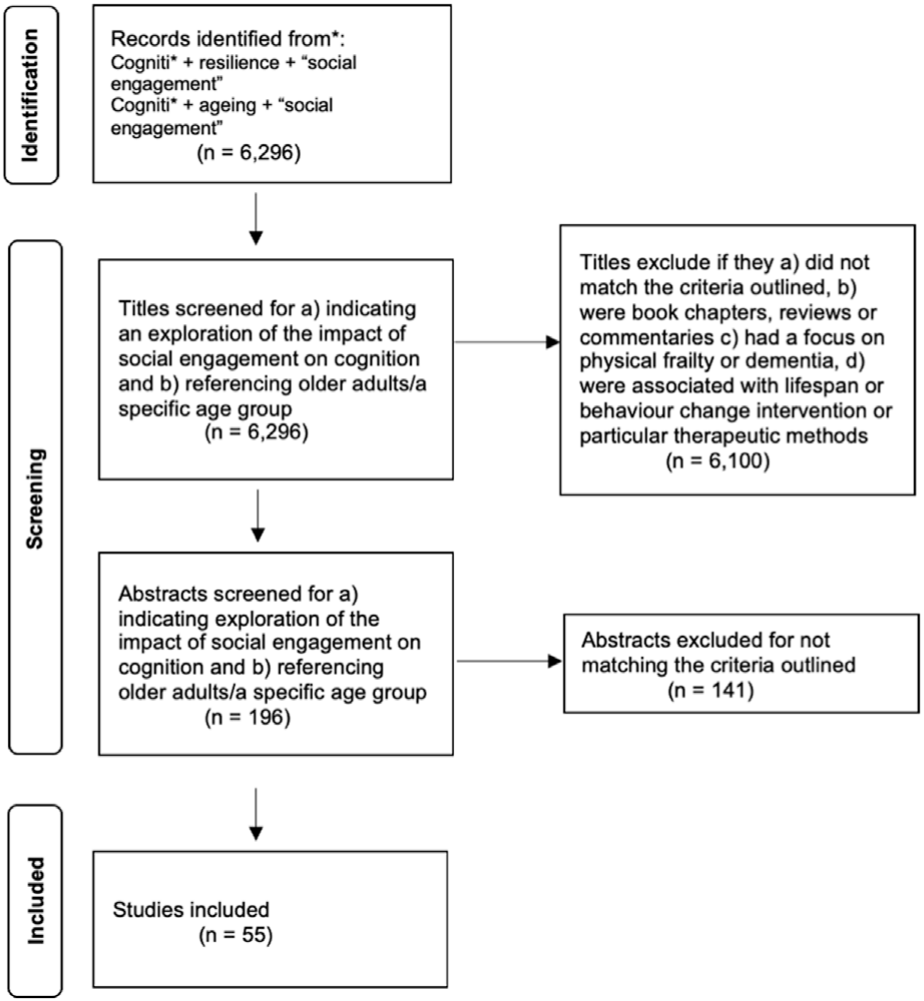
PRISMA Diagram [29] (2023, UK).

Overall, 6,296 titles were found in the literature, of which 6,100 were excluded after the title screening. The title and abstract screenings were undertaken by CB and SFD. The abstracts of the remaining 196 studies were screened using the same inclusion criteria, at which point a further 141 were excluded leaving 55 studies included in the analysis. All studies were quantitative. The PRISMA diagram (see Figure 1) identifies the outcome of screening and selection of papers.

### Framework Analysis

Four categories were derived from the analysis of collated papers, with some papers reporting results on more than one category [28]. The intention was to differentiate the opportunities for sustained social engagement and cognitive resilience or risk of cognitive decline, as indicated in the outcomes for different groups of older adults. The named categories suggest different context in which older people achieve social engagement and the relative effects on cognitive frailty. These can be defined as follows:

• *Participation* refers to participant’s use of community based social activities and the impact this has on cognitive frailty; 22 papers.
• *Household* refers to the immediate living situation and ‘home’ circumstances of an individual; 12 papers.
• *Network* refers to the characteristics of the network in which an individual operates such as its diversity, frequency of contacts, complexity, and size; 26 papers.
• *Habitat*, includes the wider neighbourhood for example, urban or rural context as a place of residence; six papers.

The tables selected for analysis are charted in Table 1. This provides information about which category the study falls in as well as the country of origin and type of study.

**TABLE 1:** Scoping review, cognitive frailty and social environment, country of origin, type of study and study categorisation (2023, UK).

Reference	Country of Origin	Type of Study	Participation	Household	Network	Habitat	Gender	Culture/racial
[30]	United States of America	Cross-sectional	x					
[31]	South Korea	Longitudinal	x					
[32]	United States of America	Longitudinal			x			
[19]	United States of America	Longitudinal			x			
[33]	Multi-national (Austria, Belgium, Czech Republic, Denmark, Estonia, France, Germany, Greece, Hungary, Ireland, Israel, Italy, Netherlands, Poland, Portugal, Sweden, Slovenia, Spain, Switzerland)	Longitudinal	x					
[34]	China	Longitudinal	x					
[35]	Ireland	Cross-sectional	x		x			
[36]	The Netherlands	Longitudinal			x			
[37]	United States of America	Cross-sectional				x		
[38]	United Kingdom	Longitudinal	x	x				
[39]	China	Longitudinal	x		x			
[40]	Canada	Longitudinal	x					
[41]	China	Longitudinal	x				x	
[42]	China	Cross-sectional			x			
[43]	Sri Lanka	Cross-sectional	x					
[44]	Taiwan	Longitudinal	x	x	x			
[45]	Denmark	Longitudinal		x				
[46]	United Kingdom	Longitudinal			x			
[47]	United States of America	Longitudinal			x			
[48]	Germany	Longitudinal			x			
[49]	United States of America	Cross-sectional	x		x			x
[50]	Mexico, United States	Cross-sectional		x				x
[51]	United States of America	Longitudinal			x			
[52]	United States of America	Longitudinal	x					
[53]	South Korea	Longitudinal		x				
[54]	South Korea	Longitudinal			x			
[55]	Spain	Longitudinal			x			
[56]	United States of America	Longitudinal				x		
[57]	South Korea	Longitudinal	x				x	
[58]	South Korea	Survey			x		x	
[59]	China	Longitudinal		x				
[60]	South Korea	Longitudinal	x				x	
[61]	Canada	Cross-sectional	x					
[62]	China	Cross-sectional	x		x			
[63]	United States of America	Cross-sectional			x			
[64]	United States of America	Longitudinal			x			
[65]	South Korea	Longitudinal	x	x				
[66]	United States of America	Longitudinal			x		x	
[67]	United States of America	Longitudinal	x		x			
[68]	Singapore	Cross-sectional		x				
[69]	Israel	Cross-sectional			x			
[70]	United States of America	Cross-sectional			x			
[71]	United States of America	Longitudinal	x			x		
[72]	Japan	Longitudinal	x				x	
[73]	Mexico	Longitudinal			x		x	
[74]	Finland, Italy, Netherlands	Longitudinal		x			x	
[75]	Germany	Longitudinal			x			
[76]	China	Longitudinal		x				
[77]	China, India	Cross-sectional		x				
[78]	China	Cross-sectional				x		
[79]	China	Longitudinal				x		
[80]	China	Longitudinal		x	x			
[81]	United States of America	Longitudinal				x		
[82]	China	Longitudinal	x					
[83]	Spain	Longitudinal			x		x	

Some evidence suggests that the social categories influence each other and that categories are not mutually exclusive in their effect on an individual [79]. But the differentiated contexts demonstrate how different environmental circumstances mediate opportunities and limitations to social engagement and enable and sustain cognitive health. The findings are in four categories and discussed below but there are some clear overarching benefits to older adults who manage to sustain social engagement. Social functioning is positively related to an initial level of cognitive function and a reduced rate of cognitive decline [32]. Social engagement protected against cognitive decline [40], and memory and executive function was predicted by social participation [33]. Conversely, not engaging socially had a negative impact on cognition. Social disengagement predicted the risk of cognitive decline in older individuals [83], and boredom from a lack of social engagement was associated with reduced cognitive function [35]. Further, demographic characteristics such as gender and culture intersect with environment or context [74].

### Participation

Participating in social activity, whether of religious or leisurely nature, including hobby and sport groups, had a positive association with cognitive functioning [34, 41, 43, 57, 67, 61 and 84]. One study specifically found that the rate of global cognitive (calculated based on a test battery that included episodic memory, semantic memory, working memory, perceptual speed and visuospatial ability) decline was reduced by 70% for those frequently active compared to those infrequently active [52, 39]. Participation in one or two social activities improved performance on cognitive tasks compared to those who participated in no social activities, and, if this was increased to three or more activities per week, they performed even better [44]. Maintaining a socially-active lifestyle enhanced cognitive reserve and benefitted cognitive function [38]. A change from no social participation to more variety in social participation was associated with improvements in cognitive function [85]. Engagement in more social activities was associated with better baseline cognitive function, though this effect disappeared over time [86].

Conversely, little participation in group activities was related with cognitive impairment [62], not being part of a senior centre was associated with decreased cognitive function [65], and infrequent participation, poor social connections and social disengagement predicted the risk of cognitive decline in older individuals [83]. Additionally, participants were found to be at higher odds for self-diagnosed memory problems if they had low cultural participation and low social engagement [30]. Thus, there is consistent evidence that participating in socially engaging activities has a positive association with cognitive functioning for the individual.

### Household

Marriage, co-habitation and home-based relationships and the loss of relationships appears to be contested and strongly influenced by gender. Living alone was not associated with poorer cognitive function at baseline and follow-up [38]. In line with this, older adults who transitioned to living alone (due to death of or divorce from spouse) had a lower risk of cognitive impairment, compared to those who continued to live with others [59]. However, men who lived alone in old age were twice as likely to experience cognitive decline compared to those married or living with someone [74], (see detail on gender influence below). Living arrangements were found to be one of the factors more strongly associated with cognitive ability in older people [45]. Older people living in multigenerational family household often had poorer cognitive function than those living with a spouse or non-relatives [59]. Data collected in Taiwan found that social participation outside the family may have a larger effect on cognition than the social contracts within the family [44, 68]. While individuals did experience more isolation from family and more loneliness than those living with others, they were often more engaged in social activities.

Being married or the presence of a partner may also affect cognition. Unmarried individuals had lower cognitive function than married individuals [76], and married participants experienced less cognitive decline [46]. Never married or divorced individuals were more disadvantaged compared to married individuals when it came to cognitive health [53]. During a nine-year follow-up, married participants had a 16% lower risk of developing cognitive impairment compared to those widowed; however when controlled for all types of social support this was no longer significant [80]. Older, single Chinese men had poorer cognitive function compared to those married, an association not found in women [77]. Another study reported that marital status was also a significant predictor of cognitive ability in women [65].

### Network

There seems to be clear evidence that a diverse or complex social network has a positive association with cognition. A diverse social network lowered the risk of cognitive decline and promoted health and survival [54]. Some evidence suggested that enhanced complexity had a buffering effect on cognitive decline [36]. Good quality social networks seem to protect against cognitive decline [63], and a higher number of networks had a positive correlation with initial cognitive function and reduced rate of cognitive decline [32].

The characteristics of the networks and their relative value to an individual seem to influence cognitive functioning. Generally speaking, having an ‘adequate’ network was associated with less cognitive impairment [62], a lower risk of cognitive decline [39], and structural elements (being married/partnered, the number of social networks and contact frequency) and functional elements (support, strain, loneliness) were associated with episodic memory [51]. However, the results regarding network size are mixed. While one study found worse perceptual speed associated with larger social networks [67], another reported that a larger network was associated with better perceptual speed and verbal fluency [75]. Individuals with no social ties were at increased risk of cognitive decline compared to those with five or six social ties [19]. Some studies also found no effect of network size on cognition [64].

The association of cognition with presence of family in an individual’s social network provided mixed results. One study found that a higher frequency of visual contact with family was associated with a lower probability of cognitive decline [83], but other studies found that relationships with family [42] and children’s visits [80] could be associated with an increased risk of cognitive impairment. Social participation outside the family network may have a larger impact on cognitive function [52], and networks with a greater proportion of friends were associated with better global cognition compared to those with a greater proportion of family [70].

More frequent contact with networks seems to have positive association with cognition [39, 69]. Alternatively, not being in a social network and experiencing social isolation a generally been to have a negative association with cognitive functioning [47, 48 and 55]. Loneliness associated with social isolation had also consistently been linked to lower cognitive functioning [46, 55], though one study reported it to be associated with better semantic memory [67].

### Habitat

One study explored how living in rural or urban places, as well as migration, influences cognition in Chinese and Indian samples [87]. For women, living in rural areas or migrating from rural-to-rural areas was associated with poorer cognition in both samples. For men in China, rural residents had worse cognitive functioning than urban residents, and those who migrated from an urban area to another urban area had the highest cognitive scores. In India, men had the poorest cognition in the migrated rural-to-rural sample. Women were consistently affected worse by migration in terms of cognitive function, compared to men, in both countries. Living in a town was associated with worse cognitive function than living in a city, and living rurally was associated with worse cognition than living in a town [79]. However, it is important to note that the characteristics of cities, towns and rural areas will differ widely between countries and these findings will not necessarily translate to all countries.

Neighbourhood characteristics also have an impact on cognition. Older Hispanic/Latina women living in a neighbourhood with the lowest perceived problems had higher global cognition and memory [37]. In the study, high perceived social cohesion in the neighbourhoods was associated with lower global cognition, as well lower verbal learning, verbal fluency and processing speed. No association between men and any perceived neighbourhood characteristics were found. Other research found that living in a cohesive neighbourhood promoted social activities and through this benefited cognitive function [86]. Neighbourhood social cohesion was also independently associated with most domains of cognitive function [81].

### Other Factors Associated With Cognition

The research evidence suggests that the different categories intersect and even contradict one another. For example, living in a cohesive neighbourhood promotes social activities and therefore benefits cognitive function, suggesting that habitat level has an influence on social participation [86]. Similarly, poor housing conditions had a negative impact on cognition because a place of residence mediates social participation [79]. A small social network also limits participation [56] although living alone can be advantageous where enabled by cultural cohesion and social activity beyond the family that builds cognitive reserve [38].

Experiencing a loss, and the connected psychological effect, has been associated with a modest decline in cognitive function [50] and loneliness is associated with network [34, 67 and 84] and household [43]. Another study found that, while loneliness is associated with aspects of cognitive ability, symptoms of depression account for this association [61], suggesting that presence of depression symptoms may be more significant for cognition than loneliness.

### Gender Differences

The impact of social engagement, as well as the type of social engagement and its connection to cognition, seemed to differ between genders, though the research is not yet conclusive. One study found that volunteering significantly aids cognitive functioning in women but not in men [41]. While religious group activities were found to be beneficial for women but not men, an inability to work was detrimental to men’s cognition but not for women [57]. Additionally, social activities were likely to maintain good cognitive functioning in older age for men, and for women with lower education, social engagement particularly protected against cognitive decline [60]. Another study found that men in neighbourhood associations and local events groups and women engaged in hobby and volunteer groups experienced less cognitive decline [72].

Women’s cognition was influenced by the number of members they considered friends [58], and their engagement with friends was protective against cognitive decline [83]; this was not found in men [83]. For men, high perceived support at baseline was associated with increased risk of cognitive impairment, an effect not found in women [66]. Additionally, for women who remained in Mexico while their children emigrated to the US, this led to a stronger decline in overall cognitive performance whereas there was no impact on men [73].

As mentioned above, men who lost a partner, were unmarried or started to live alone in old age had at least two times subsequent stronger cognitive decline compared to those married or living with someone [74].

### Racial and Cultural Differences

Racial and cultural demographics and differences also impact cognitive decline. Cultural effects of social participation were also found where social participation was positively associated with memory in non-Hispanic White peoples but not in non-Hispanic Black peoples [49]. This may suggest some cultural groups are less likely to participate in enough social activity to yield cognitive benefits.

Cultural and gender effects were also found when it came to comparing the cognition of wives in Mexico and the United States. In Mexico, the wives’ social engagement benefited their own cognitive function, as well as their husbands, and this effect was found this direction only. In the United States, no effects of one’s spouse engagement were found, from which the authors suggest that if there is a more traditional social role of women there might be more co-dependency within the couple [50].

### Strengths and Limitations

This study used a single search engine to scope a vast literature and categorise environmental factors that promote social engagement. It does not reflect the literature related to loneliness as a key indicator of cognitive frailty. Loneliness as a personal psychological phenomenon has been widely studied and the negative effects on cognitive health and wellbeing are associated not with isolation but rather the perception of being alone.

Similarly, this scoping review does not address historic socio-environmental factors. For example, some evidence suggests that childhood connections have an impact on cognition in later life, with adverse childhood events and negative friendship experiences significantly associated with lower initial cognitive status and the rate of decline in cognitive functioning [52].

The search did not specifically include “frailty” for reasons described in the methods section. The inclusion of English only studies presents a risk of a dominant Eurocentric view in spite of international cohorts included and analysed.

## Discussion

There are multiple correlates of cognitive ability level and change in later life, many of which have small effects [88]. But a cumulative effect of small environmental improvements in old age may significantly mitigate the negative effects of sparse social contact without necessitating complex social or lifestyle behaviour change interventions that are difficult and costly to deliver through services [89].

**FIGURE 2:**
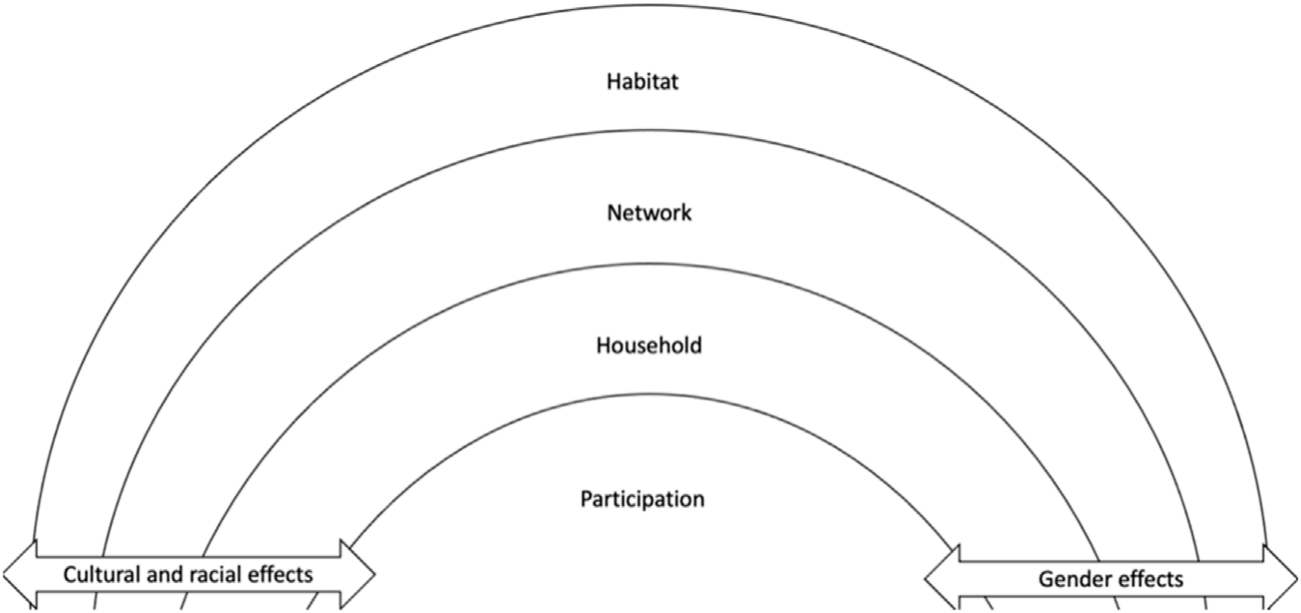
Synthesis of environmental factors associated with cognition in older adults (2023, UK).

An initial framework for considering the factors that mitigate the risks to cognitive decline in very limited social environments is represented in Figure 2. It may offer a useful way to think about and promote an approach to planning more socially enabling environments, particularly for those in the most deprived and vulnerable communities.

WHO use the term “participation” within the International Classification of Functioning (ICF) as a person’s involvement in a life situation and involves engagement in life roles, such as employment, education, or relationships [Disability and Health Overview | CDC]. It is suggested that technology, support and relationships, services, policies, or the beliefs of others all reflect in participation achieved within an environment. The environment informs and sometimes compensates for individual characteristics including municipal or household measures to compensate for ageing.

This evidence suggests that the presence of family support (for example, personal care in a multigenerational family home) is insufficient to prevent cognitive decline. The studies undertaken in Asia, where family care is more pervasive demonstrates that care, in and of itself does not prevent cognitive frailty [53]. The absence of friendship, engagement beyond an immediate family group and the inability to participate with neighbours, acquaintances and in group activities can be a cause or a consequence of cognitive decline and may have a causal relationship in older adult populations.

There are cross-cutting drivers associated with environmental factors including gender effects; men appear to be more specifically affected by community and household isolation, irrespective of household, providing there is access to external social networks [76, 48]. In addition, cultural differences, particularly associated with the global diversity of the literature can be seen, with different norms in relation to expectations of families to offer care and support for older relatives. The absence of information about the effects of migration, multi-cultural neighbourhoods and the impact on social networks is evident, with this being a much more pervasive situation in poorer communities and requiring specific development at community level.

The presence or absence of an “anchor organisation” (church communities, charity and third sector organisations) at community level, may be important to address local participation, and facilitate population health and wellbeing. These organisations operate in neighbours with specific knowledge of critical cultural factors, enabling local areas to challenge the difficulties associated with social isolation. Cognitive frailty may be particularly prone to social avoidance, if safety, comfort and practical requirements are inadequate and fail to compensate for personal disability [90]. Further investigation is needed into the likelihood of cognitive frailty given cognitive decline [91].

## Conclusions

This review investigated how older people experience cognitive outcomes in relation to context. The analysis presents a complex and diverse range of influences on cognitive health and frailty.

The paper demonstrates that contextual factors should be a consideration for older people and that there is a need to address vulnerability to pervasive cognitive frailty due to isolation and sub-optimal opportunities for social connection and engagement.

Interventions that constitute a community-based prevention strategy for those at risk may be indicated. Health and social care commissioners and town planners may consider cognitive frailty as a specific population health risk and apply wide-ranging asset-based development programmes to enable communities to address the critical needs across neighbourhoods.

Funded and conceived of within the Cognitive Frailty Network (CFIN), the project aligns to further investigations of air quality and access to green space that are also associated with risks to cognitive decline in older adults.
